# What Arguments against COVID-19 Vaccines Run on Facebook in Poland: Content Analysis of Comments

**DOI:** 10.3390/vaccines9050481

**Published:** 2021-05-10

**Authors:** Dominik Wawrzuta, Mariusz Jaworski, Joanna Gotlib, Mariusz Panczyk

**Affiliations:** Department of Education and Research in Health Sciences, Medical University of Warsaw, 02-091 Warsaw, Poland; mariusz.jaworski@wum.edu.pl (M.J.); joanna.gotlib@wum.edu.pl (J.G.); mariusz.panczyk@wum.edu.pl (M.P.)

**Keywords:** Facebook, vaccine hesitancy, vaccine, COVID-19, infodemiology

## Abstract

Social media allow anti-vaxxers to quickly spread misinformation and false statements. This situation may lead to an increase in vaccine hesitancy. We wanted to characterize what arguments against COVID-19 vaccines run on Facebook in Poland. We analyzed Facebook comments related to the five events of the introduction of COVID-19 vaccines—announcements of the efficacy of the Pfizer-BioNTech (09.11.2020), Moderna (16.11.2020), and AstraZeneca (23.11.2020) vaccines, registration of the Pfizer-BioNTech vaccine by the European Medicines Agency (21.12.2020), and the first vaccination in Poland (27.12.2020). We collected the comments from fanpages of the biggest Polish media and then established their main anti-vaccine themes. We found that the negative arguments about COVID-19 vaccines can be divided into 12 categories. Seven of them are universal and also apply to other vaccines but five are new and COVID-19’ specific. The frequency of arguments from a given category varied over time. We also noticed that, while the comments were mostly negative, the reactions were positive. Created codebook of anti-vaccine COVID-19 arguments can be used to monitor the attitude of society towards COVID-19 vaccines. Real-time monitoring of social media is important because the popularity of certain arguments on Facebook changes rapidly over time.

## 1. Introduction

### 1.1. Background

Social media provide great opportunities for sharing convictions and opinions. Unfortunately, there is also a place for conspiracy theories. Almost half of the health content published on social media contains misinformation [1]. The subject of vaccinations and infectious diseases is, next to oncological topics, the most frequently misrepresented in social media [2]. For these reasons, social media are currently regarded as the main communication tool for people with anti-vaccine attitudes [3,4].

An example of an uncontrolled spread of misinformation on vaccination on social media is the measles outbreak in Samoa. In July 2018, two Samoan children died after MMR vaccination because nurses inadvertently used curare instead of water to dilute the MMR vaccine. This resulted in an increased activity of local anti-vaxxers on Facebook, who took action to discourage vaccination [5]. As a result, measles vaccination coverage in Samoa decreased to 31% [6]. Consequently, measles outbreak occurred on the islands in September 2019, causing over 5000 cases and 83 deaths in a country with the population of 200,000 [7]. Finally, the authorities decided to organize a vaccination campaign and arrest some anti-vaccination campaigners spreading misinformation on Facebook [8]. When 95% of eligible people in Samoa got vaccinated, the outbreak was eliminated [9]. Another case happened during the COVID-19 pandemic when European trust in AstraZeneca’s vaccine plummeted after spreading information on rare episodes of blood clotting after the vaccination [10]. This decline in confidence has occurred in spite of the European Medicines Agency’s statement that the overall benefit-risk of this vaccine is positive and that blood clots are a very rare side effect [11].

These cases show that negative online information on vaccination is one of the leading causes of vaccine hesitancy [12]. The Internet has a huge impact on society’s decisions as 70% of Internet users make decisions about vaccination on the basis of information found there [13], which discourages almost half of the parents [14]. Even a few minutes of contact with anti-vaccine information on the Internet negatively affects the perception of vaccine-related risks [15] and reduces parents‘ willingness to vaccinate their children [3]. Recently, Facebook and Instagram have taken steps to stop the spread of misinformation, making it difficult to be found. Unfortunately, this type of content has still not been completely removed from these platforms [16,17].

When we analyze online comments on vaccinations, attention should also be paid to the features of social media. Facebook’s user-generated content is more difficult to obtain and analyze than Twitter’s content, because this platform does not provide an application programming interface (API) allowing to automatically search and retrieve data. Despite these obstacles, Facebook can provide much more accurate data on public opinion. In comparison to Twitter or Instagram, Facebook has not only much more active users, but the demography of its users is similar to whole society‘s demography. Twitter and Instagram have an overrepresentation of young, well-educated, and living-in-cities users [18].

Anti-vaccine social media content is especially dangerous because it is often professionally created and catches the audience‘s eye. Authors of such content use celebrities and personal stories talking about negative effects of vaccines [19], but their content is rarely created by health care professionals [20,21]. They underline side effects and vaccination risks causing fear in their audience [22,23]. This tactic is based on behavioral research showing that fear-related information attracts audience attention [24]. Additionally, almost a quarter of anti-vaccine social media content contains conspiracy theories [25]. The effect of all these characteristics is the high popularity of anti-vaccine content, often viewed and shared by social media users [22,26,27].

During the COVID-19 pandemic, vaccine hesitancy may delay the combat of SARS-CoV-2 virus despite vaccines’ quick development [28]. The studies conducted so far indicate that not all social groups want to be vaccinated equally willingly [29]. Surveys show that about a quarter of Europeans do not want to take COVID-19 vaccine [30]. Therefore, in order to take full advantage of the great scientific success, it is necessary to study public opinion, because trust in science and the general attitude towards vaccines’ efficacy have an impact on the willingness to vaccinate against COVID-19 [31].

### 1.2. Aim

We wanted to find out and categorize negative public opinions on COVID-19 vaccines. To achieve this goal, we tried to categorize the content created by Facebook users. We focused on comments published in the response to the news published on Polish media fanpages, describing COVID-19 vaccines’ development.

In our research, we seek to answer three research questions:

RQ1: What arguments do Facebook users employ against COVID-19 vaccines? Have they created new anti-vaccine arguments specifically connected to this pandemic and not existing before in the context of other vaccines?

RQ2: How does the popularity of arguments against COVID-19 change over time?

RQ3: What reactions on COVID-19 vaccines do Facebook news receive? How have these reactions changed over time?

## 2. Materials and Methods

### 2.1. Data Collection

To collect data, we created a list of the most popular Polish media by the number of users (newspapers, TV stations, radio stations, news websites). For each medium, we found an official Facebook fanpage. Finally, we had a total of 14 fanpages in our dataset. We present an overview of the analyzed media in Table 1.

In the next step, from 1 November 2020, we started to look for highly-commented events in Poland, related to COVID-19 vaccines. The authors decided by consensus which events should be included in the study. We planned to end our observation when the first person would be vaccinated in Poland.

As a result, we added five highly-commented events in Poland to our analysis, which are described in Table 2. The first news was three week-by-week events related to the end of the vaccine research. Pfizer-BioNTech was the first to announce the completion of the research, followed by Moderna and AstraZeneca. The next event was the registration of the Pfizer-BioNTech vaccine by the European Medicines Agency (EMA). Finally, we analyzed the comments connected to the news about the first vaccination in Poland.

We conducted the same analysis for each event. One week after the event, when the number of new comments and reactions on social media typically falls sharply [32], we collected data. We searched for a post describing the analyzed event on every fanpage from our list. Secondly, we downloaded all comments (using the tool exportcomments.com, accessed on 1 May 2021) and all the reactions connected to this post. In summary, we collected 22,791 comments for further analysis.

### 2.2. Themes of Comments

We wanted to create a codebook of categories of Facebook opinions against COVID-19 vaccines. Firstly, from our comments dataset, we randomly chose 1000. Then, we decided to use the list of categories of vaccine-related social media comments proposed by Broniatowski et al. [33] as a base set of topics to which we tried to assign each of the comments. Two researchers (DW and MP) independently labeled every comment with one category. If there was no proper category in our primary set, we created a new category. Finally, we established by consensus the codebook with 12 main themes of anti-COVID-19 vaccines comments.

### 2.3. Data Analysis

From each post, we chose 15% most-reacted comments for further analysis. In the next step, five researchers wanted to assign those 3414 comments to categories from our codebook.

Firstly, we assessed our interrater reliability. We got 50 random comments and each of evaluators (DW, MP, JG, KK, MJ) independently assorted each comment into one category. Then, we calculated the value of Krippendorff‘s Alpha [34] to estimate interrater reliability among coders, finding that our interrater agreement was high (α = 76%).

As there was a large consensus on our assessments, we decided to evaluate the comments individually. We divided our dataset of 3414 comments into five parts, with one researcher coding 682 comments, and the other four coding 683 comments each.

## 3. Results

### 3.1. Data Overview

In Table 2, we present all events included in our analysis. For each event, we show the date of news publication, the number of comments, reactions, and shares in all included media. Besides that, we display a column with the number of media which posted the information, because not all media reported every event. The first vaccination in Poland was the most commented event and also got the most reactions. The second most popular event was the first information at the end of vaccine research.

### 3.2. Research Question 1

We found that comments on COVID-19 vaccines can be divided into 13 categories, 12 negative and 1 positive, presented in Table 3. Seven categories are similar to general themes of anti-vaccine social media posts, previously described in the literature [33]. Five themes are new and specific to COVID-19 anti-vaccine sentiment. They argue that these vaccines have been developed too quickly without a proper test. They also suggest that vaccine does not exist despite the information from pharmaceutical companies and governments. Some users remind about the development of the swine flu vaccine. This vaccine was also developed quickly, but, over time, it caused more side effects than expected. On the other hand, conspiracy theories suggest that vaccines against COVID-19 existed before the official announcement, even before the pandemic. The last specific argument implies that people should avoid vaccination because no one takes responsibility for the potential side effects of the vaccine. Only 15% of the comments were positive, while 85% were negative.

### 3.3. Research Question 2

**Figure 1:**
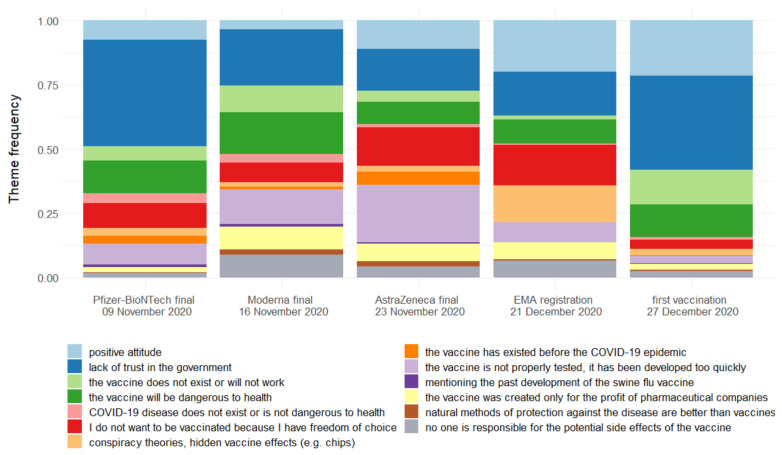
Distribution of themes.

In this part of our research, we checked the popularity of topics found in the previous step. The most popular anti-vaccine arguments in our dataset are: the lack of trust in the government, the danger of vaccines, and the lack of faith in the existence of an effective vaccine. We can notice that a share of pro-vaccine comments has been growing over time. It has increased from 7% to 22% during the event of first vaccination. Figure 1 shows the distribution of themes over time.

In the first three events informing about the completion of vaccine studies, there are visible doubts about the vaccine being developed too quickly and not adequately tested. With the completion of studies of the second and the third new vaccine (Moderna, AstraZeneca), these doubts grew. Regarding the completion of research on the Moderna and AstraZeneca vaccines, there are also a lot of comments suggesting that the vaccines were created only for money. After European Medicines Agency (EMA) registration of Pfizer-BioNTech vaccine, a fear of improper testing has faded away. Then, the focus shifts to convictions that the vaccine is not working.

The first and last events have a similar topics’ distribution, especially regarding a high aversion to the government. On the other hand, after the EMA approved the Pfizer-BioNTech vaccine, we have a lot of conspiracy theories and little criticism of the government.

### 3.4. Research Question 3

**Figure 2:**
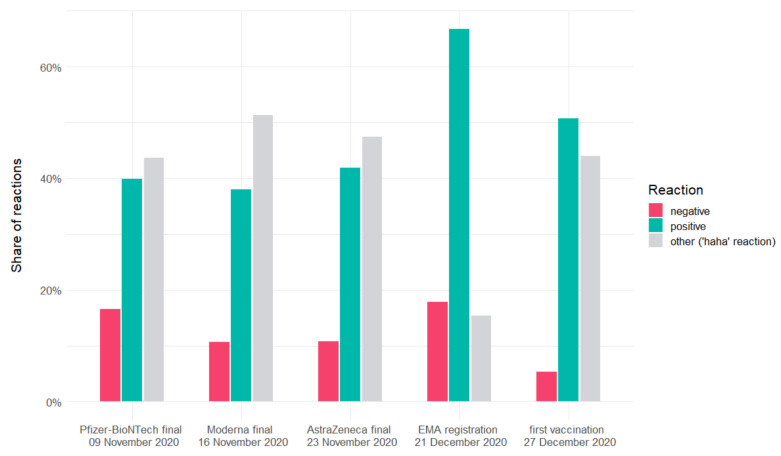
Reactions connected to the news.

Figure 2 shows Facebook users’ reactions to the news. We introduced reactions such as ‘like’, ‘love’, ‘care’, and ‘wow’ in the positive group and reactions such as ‘angry’ and ‘sad’ to the negative group. Reaction ‘haha’ was classified as ‘other’. We referred to the findings by Giuntini et al. [35] for this classification, describing Facebook reactions’ sentiment. The positive-to-negative-reactions ratio has increased over time. It is interesting that positive reactions prevailed over negative ones, while the comments remained strongly negative.

## 4. Discussion

### 4.1. Overview

We found that negative COVID-19 vaccine comments can be divided into 12 categories. Seven of these arguments are universal and also apply to other vaccines, but five types are new and COVID-19-specific. New topics say that the COVID-19 vaccines do not exist or work, are not properly tested, have existed before the epidemic, can be dangerous as previous flu vaccine, or no one is responsible for potential side effects.

Our codebook of anti-vaccine COVID-19 arguments can be used by public health specialists and researchers to monitor society’s attitude to COVID-19 vaccines. Real-time social media monitoring is important because we have found that popularity of certain arguments on Facebook changes rapidly over time.

### 4.2. Main Topics of Anti-Vaccine Comments

The most common arguments against COVID-19 vaccines are based on the lack of trust in the government, which is responsible for organizing the vaccination programs. This is consistent with previous studies, showing that low trust in the government is related to vaccine hesitancy [36]. It is important to pay attention to the role of governments in the communication of vaccination [37]. Satisfaction with the government can influence vaccination beliefs and willingness to get vaccinated [38]. This thread requires further comparative research in other countries, as OECD data shows that Poland’s citizens generally have one of the lowest levels of trust in the government in Europe [39].

Another anti-vaccine comments’ topic suggests that COVID-19 vaccines are dangerous to health. It is one of the most common arguments used by anti-vaxxers to create fear [40]. This topic is especially dangerous when we understand that emotional stories and fear-related information highly attract the audience’s attention [24,41]. Other general arguments mention that infectious diseases (in this case COVID-19) are not dangerous, or that natural methods of obtaining immunity are more effective.

Facebook users often express the opinion that they do not want to be vaccinated because of a free choice that no one should limit. It can be connected to the recent research showing that people have a reduced risk perception of being ill with COVID-19 [42]. Some of the comments deal with conspiracy theories. Most often, they contain general arguments not strictly related to the COVID-19 vaccine, such as microchips in vaccines. Generally, anti-vaccine social media content often presents unverified information [22]. In the case of COVID-19, anti-vaccine users claim that vaccines were developed only for the pharmaceutical corporations’ profit, and not for society’s health. This argument is interesting in the context of AstraZeneca’s announcement that it will not profit from the COVID-vaccine [43].

Facebook users also employed specific arguments against COVID-19 vaccines that have not been common in the anti-vaccine discourse so far. Many accuse the vaccine of being developed too quickly and without a properly test. These doubts are most likely caused by the speed of clinical trials and registration [44]. It is important for public health authorities to publicly emphasize that vaccines registered in the European Union and the USA have undergone the entire registration process [45,46]. Anti-vaccine commenters often mention the fast development of the swine flu vaccine. They underline that this vaccine was created very quickly and presented as safe, like the COVID-19 vaccine, but, in Sweden, increasing cases of narcolepsy were reported after vaccination [47].

Another theme suggests that no one is responsible for vaccines’ side effects. This is a false because European governments set up a compensation fund to offer payments to people suffering with severe side effects after vaccination [48,49]. Some people say that COVID-19 vaccines existed before the COVID-19 pandemic. This claim could be based on the popular conspiracy theory suggesting that the virus was purposefully created and spread [50]. The last specific anti-vaccine argument says that the vaccine probably does not exist, and all information about efficiency and successful registration is fake. This can be connected with a conspiracy theory that COVID-19 was created as a biological weapon [51].

### 4.3. Changes in Content Over Time

Five researchers categorized comments. To assess their interrater reliability we used Krippendorff’s Alpha method which allows the assessment of agreement between more than two researchers. The calculated alpha value was 76%. According to the article published by Krippendorff, conclusions are acceptable when α >= 0.667 [52]. In our case, some discrepancies in the assessment were caused by the ambiguity of the comments. In some of the comments, it was not clear whether the user was making a negative argument or if was in favor of vaccinations, but the comment was ironic. In such situations, there could be a discrepancy in the assessment between the evaluators. Previous studies have shown that without paralinguistic cues like gestures or intonation it can be difficult to detect sarcasm in online messages [53,54].

Over time, the number of positive comments about vaccination against COVID-19 has increased. The reasons for this situation are multifactorial. On the one hand, trust grows with time [55]. History shows that new technologies were often not trusted at the time of their creation, before society understood the benefits and risks of a given technology [56]. Additionally, media systematically presented the vaccine as a great success, building trust and willingness to vaccinate. In December, Polish public health authorities conducted education campaigns to convince society that COVID-19 vaccines are effective, safe, and properly tested.

We also found that shares of negative themes vary over time. During the news of the completion of the vaccine research, there were many doubts in the comments about fast vaccine development and concerns about proper testing. This is reinforced by the fact that information about the completion of three studies appeared within 2 weeks after just 9 months of development [28,57,58]. Considering the complexity of the process, obtaining results so quickly could cause public distrust. These concerns were widely commented on by public health experts, who explained that the clinical trial process had passed all necessary formalities [59]. An example of the early undertaken action in response to popular anti-vaccine arguments is the activity of the Polish National Institute of Public Health. In December 2020, after the announcements of the efficacy of the vaccines, many comments on Facebook were saying that the vaccines are not properly tested. In the response to these doubts, the Institute started an action on social media explaining what the clinical trials of vaccines involve. This could potentially lead to the reduction of the popularity of the argument that the vaccines were developed too quickly. Our study shows that such a decline was visible at the end of December. The theme of the comments began to change in the direction that the vaccines do not work. Besides, in our comments’ dataset, many users misinterpreted the vaccine efficacy data, suggesting for example that 90% efficacy means that 10% of patients have severe side effects. Data misinterpretation is a common tactic by anti-vaccine movement to affect vaccine decisions [60].

The positive-to-negative-reactions ratio has increased over time. One of the reasons may be a better understanding of the situation and vaccines’ characteristics. Over time, recipients have had the opportunity to read a variety of information and dispel some of their doubts. At that time, the Polish public health authorities conducted information campaigns that could have caused this effect [61]. There is also a discrepancy between the sentiment of the comments and the sentiment of the reactions. A vast majority of comments are negative, while the reactions are mostly positive. This is in line with the research by Yang et al. [62], who found that Facebook users publish two times more negative than positive comments on fanpages.

Currently Facebook users can use 7 reactions (‘like’, ‘love’, ‘care’, ‘wow’, ‘angry’, ‘sad’, ‘haha’) which allow them to react to posts with emoticons. Such a large number of available reactions is a relatively new phenomenon, it was introduced on Facebook in 2016. So far, there have not been many papers exploring how users use these animated reactions. One of the first comprehensive attempts to identify emotional expressions of Facebook reactions was made by Giuntini et al. [35]. According to their results, we classified ‘like’, ‘love’, ‘wow’, and ‘care’ as reactions used by COVID-19 vaccines supporters. We categorized the ’sad’ and ‘angry’ reactions as used by people reluctant to COVID-19 vaccines. The unambiguous assignment of the ’haha’ reaction was problematic. This reaction should be used in response to funny content. In practice, as demonstrated by Giuntini et al. [35], it is not related to any specific emotion and is used in a variety of contexts. Facebook users use this reaction as a positive, negative, or neutral with similar frequencies. Since it is impossible to determine the exact sentiment of the ‘haha’ reaction, we assigned it into the ‘other’ category.

## 5. Conclusions

The development of COVID-19 vaccines has prompted new arguments among anti-vaxxers. They accuse the vaccines of not being properly tested and safe. However, their main doubt is a classic argument that they do not trust the government which is responsible for vaccination campaigns.

The comments regarding vaccination have an overwhelmingly negative sentiment, but the reactions are positive. This suggests that pro- and anti-vaccine groups have different patterns of dealing with social media content. This is an important finding because many researchers analyze only the sentiment of Facebook reactions without focusing on the comments.

Our codebook of anti-vaccine topics, presented in Table 3, can help public health professionals to better understand social media content. Early recognition of social doubts allows for targeted information campaigns. Additionally, our codebook can be a contribution to future analysis by researchers and public health specialists who monitor society’s attitude towards COVID-19 vaccines.

## Figures and Tables

**Table 1:** Media included in the study.

Fanpage	Type of Media	Number of Facebook Fans
fakt24pl	newspaper	1,138,628
wyborcza	newspaper	757,197
dziennikrzeczpospolita	newspaper	135,033
wwwsepl	newspaper	122,522
radiozet	radio station	961,349
rmf24	radio station	424,580
Onet	news website	1,141,477
gazetapl	news website	796,400
wiadomosciwppl	news website	688,382
portalo2pl	news website	450,600
interia	news website	107,192
tvn24pl	TV station	1,472,732
tvp.info	TV station	401,223
polsatnewspl	TV station	239,761

**Table 2:** Events included in the study.

Event	Date	Number of Comments	Number of Reactions	Number of Shares	Number of Media that Reported
Pfizer-BioNTech announces effectiveness of the vaccine	9 November 2020	7181	9608	702	14
Moderna announces effectiveness of the vaccine	16 November 2020	2117	3522	139	13
AstraZeneca announces effectiveness of the vaccine	23 November 2020	2160	2151	135	8
registration of the Pfizer-BioNTech vaccine by EMA	21 December 2020	2017	3059	130	13
first vaccination in Poland	27 December 2020	13,254	15,626	682	14

**Table 3:** Themes of comments and examples from our dataset, new COVID-19 vaccine specific themes are in bold.

Theme	Example Comment from Our Dataset
positive attitude	I will get vaccinated as soon as there is an opportunity. I can record it and send to all those with anti-vaccine views.
lack of trust in the government	First, let the government test it on themselves and after some time we’ll see if it’s so cool.
the vaccine will be dangerous to health	The television fools you. Vaccines are death.
COVID-19 disease does not exist or is not dangerous to health	Why the vaccine if there is no COVID?
I do not want to be vaccinated because I have freedom of choice	I’m not gonna be a guinea pig.
conspiracy theories, hidden vaccine effects (e.g., chips)	It is about implanting people with chips or some other shit.
the vaccine was created only for the profit of pharmaceutical companies	They created the virus, gave it time to enter the human psyche and arouse fear, they created the demand, and now they provide the supply of the vaccine everyone wants and will pay trillions.
natural methods of protection against the disease are better than vaccines	No vaccine can give you a 100% guarantee. I prefer to naturally strengthen my immunity and not to get sick.
**the vaccine does not exist or will not work**	They will be vaccinated with saline in front of all people. They will think these are real vaccines.
**the vaccine has existed before the COVID-19 epidemic**	They had a vaccine a long time ago. Now there is too much resistance. They still have to train people to give up easier.
**the vaccine is not properly tested, it has been developed too quickly**	Normal vaccines have been produced and tested for a minimum of 8 years. And here ... less than a year and ready. I wonder what these people will go through in a few years.
**mentioning the past development of the swine flu vaccine**	There was already a swine flu vaccine invented in a few months. Poland, fortunately, did not have money for it, but Sweden pays compensation to people who got vaccinated for side effects, e.g., narcolepsy.
**no one is responsible for the potential side effects of the vaccine**	If this vaccine is so great, why are pharmaceutical companies released from liability for side effects?

## Data Availability

The datasets are available on request to the corresponding author.
